# Ibuprofen Improves Wheat Growth Under Salinity by Modulating Hormonal and Antioxidant Status

**DOI:** 10.3390/plants15030360

**Published:** 2026-01-23

**Authors:** Dilara Maslennikova, Oleg Mozgovoj

**Affiliations:** 1 Institute of Biochemistry and Genetics—Subdivision of the Ufa Federal Research Centre of the Russian Academy of Sciences, Prospect Oktyabrya 71, Lit. 1E, Ufa 450054, Russia; 2 Institute of Petrochemistry and Catalysis—Subdivision of the Ufa Federal Research Centre of the Russian Academy of Sciences, Prospect Oktyabrya, 141, Ufa 450075, Russia; skill15@mail.ru; 3 Department of Molecular Technologis, Ufa State Petroleum Technological University, Kosmonavtov Street 1, Ufa 450064, Russia

**Keywords:** ibuprofen, wheat (*Triticum aestivum* L.), phytohormones, redox status, salinity, nuclear magnetic resonance

## Abstract

Pre-sowing seed treatment (priming) is a strategic tool for programming future crop yield, aimed at improving early plant development and enhancing stress resilience. This study investigated the effects of priming wheat seeds with 100 µM ibuprofen on early ontogeny under optimal conditions and salt stress (100 mM NaCl). An evaluation of germination energy, growth parameters, phytohormone levels (abscisic acid, indolylacetic acid, and cytokinins) and the status of the antioxidant system in 7-day-old seedlings demonstrated that ibuprofen treatment stimulates wheat growth and tolerance, despite its absence of accumulation in plant tissues. Modulation of hormonal balance plays a key role in these protective effects: under optimal conditions, ibuprofen elevates abscisic acid and indolylacetic acid levels, while under salt stress, it prevents excessive abscisic acid accumulation and mitigates the stress-induced decline in indolylacetic acid and cytokinins. Furthermore, ibuprofen promotes a coordinated increase in glutathione, ascorbate, and H_2_O_2_ levels, concomitant with the activation of key enzymes (glutathione reductase and ascorbate peroxidase), thereby enhancing the plants’ antioxidant potential. Under saline conditions, ibuprofen pretreatment also reduces stress-induced dysregulation of this system. Therefore, ibuprofen acts as a hormetic preconditioning agent that improves seedling vigor and stress tolerance by fine-tuning hormonal signaling and redox metabolism.

## 1. Introduction

As a staple food crop, wheat (*Triticum aestivum* L.) is a fundamental source of calories in human nutrition. The major producers of wheat in 2022 were China, India, Russia, and the European Union, with a total global production of about 778 million metric tons [1]. Biotic and abiotic stresses represent major constraints to grain crop productivity [2]. Among abiotic stresses, soil salinity is a particularly widespread and detrimental challenge. It arises from a hydrological imbalance: irrigation water or groundwater evaporates, leaving dissolved salts to accumulate in the soil profile. This process reduces soil fertility and poses a significant threat to agricultural productivity. The problem is further exacerbated by climate change, which is contributing to the global expansion of salt-affected lands. Estimates indicate that saline soils cover roughly one-tenth of the world’s continental land area [3,4]. Salinization, along with the solonetzization process, constitutes a major land degradation issue in Russian agriculture, affecting an estimated 18.2% of the country’s total sown area (~80 million ha) [5]. It should be noted that plants are susceptible to the detrimental effects of salinity throughout their entire life cycle, but they are most vulnerable during the germination and seedling stages. Salt stress exerts both osmotic and toxic effects on plants, leading to the inhibition of vital processes and physiological functions such as photosynthesis, mineral nutrition, and growth [4]. This condition chronically impairs plant development and causes considerable losses in agricultural productivity.

Due to this, improving crop resilience through plant growth regulators is a crucial challenge for wheat cultivation. Advances in modern agronomy have led to the development of a diverse array of compounds that can induce plant resistance. Salicylic acid (SA), a recognized phytohormone and antioxidant, is well documented in the scientific literature for its ability to induce resistance in wheat [6,7,8].

Formulations based on it, such as “Immunakt-SK”, “SalicylPure”, and “Salibor”, are finding increasingly widespread application in agricultural technologies, as they effectively activate plant defense mechanisms and promote growth productivity. It is noteworthy that SA has not only served as the basis for modern agrochemicals but has also become the progenitor of an entire class of pharmaceutical drugs. Its chemical structure served as the blueprint for the development of a series of non-steroidal anti-inflammatory drugs (NSAIDs), such as the well-known ibuprofen (IBU), or 2-(4-isobutylphenyl) propionic acid [9].

According to data presented on the website https://www.chemanalyst.com/industry-report/ibuprofen-market-3151 (accessed on 10 December 2025), the global ibuprofen market volume in 2023 was approximately 46 thousand tons and is expected to grow at a compound annual growth rate of 2.3% until 2034. It is also important to note that pharmaceutical substances, including IBU, are ubiquitous microcontaminants. NSAIDs have low biodegradability and tend to accumulate, and today their concentration in the ecosystem has reached a critical level. A global assessment by Wilkinson et al. (2022) of 258 rivers in 104 countries showed that pharmaceutical pollutants at potentially toxic concentrations were detected in more than a quarter of the studied rivers across various world regions [10].

Consequently, the current ecological landscape underscores an urgent need to investigate the mechanisms governing plant interactions with environmental micropollutants. A comprehensive understanding of how plants absorb, distribute, accumulate, and metabolically respond to these xenobiotics is essential, and must be coupled with a parallel assessment of their physiological, biochemical, and growth parameters. It will provide a basis for predicting and developing methods to use them in ways that mitigate environmental risks to ecosystems, as well as for modeling their positive regulation of plant growth and development. To date, we have reason to believe that IBU regulates plant metabolism by acting as an inhibitor of jasmonate-induced processes [11,12]. This regulation likely occurs through the modulation of lipoxygenase activity in plants, which shares functional similarities with human cyclooxygenase. These effects are supported by experiments involving the foliar spray of 5 mM IBU on garden strawberry (*Fragaria* × *ananassa* Duch.) berries, as well as the supplementation of 10 µM IBU into the germination medium of rice (*Oryza sativa* L.) seeds [11,12]. The possibility of using IBU to enhance plant stress tolerance was first proposed in a patent [13]. In this patent, the authors suggest using IBU to increase the salinity tolerance of millet plants, demonstrating a good anti-stress effect through seed pretreatment and the addition of the compound to the growth medium together with NaCl. It is impressive that the anti-stress effect persists for up to 21 days of plant vegetation, with concentrations of 50 and 100 µM of the preparation showing good efficacy.

Our prior work demonstrated that 100 µM IBU modulates key physiological pathways, including those related to salicylic acid, glutathione, and glutathione reductase (GR) activity [14], and affects the photosynthetic apparatus, nitric oxide homeostasis, and antioxidant capacity [15]. Phytohormones—including indolylacetic acid (IAA), gibberellins, cytokinins (CKs), abscisic acid (ABA), ethylene, jasmonates, and salicylic acid—are small molecules synthesized by plants at low concentrations. They regulate a broad spectrum of physiological and stress-response processes, such as cell division, elongation, differentiation, flowering, fruiting, seed germination, senescence, and responses to biotic and abiotic stresses [16]. Their actions are coordinated through extensive signaling crosstalk, which allows for the fine-tuning of adaptive processes upon exposure to biotic/abiotic stresses or xenobiotic such as IBU.

Thus, the available data suggest that IBU can be considered a xenobiotic with high potential for regulating plant growth and stress tolerance. To test this hypothesis and assess the environmental safety of its application, this study aimed to address the following objectives: (a) to quantify IBU uptake and accumulation in wheat plants following seed priming; (b) to determine the involvement of key phytohormones— ABA, IAA, and CKs—in mediating the IBU response; and (c) to assess the response of the antioxidant defense system in plants grown under both normal and saline (100 mM NaCl) conditions.

## 2. Results

### 2.1. Accumulation of IBU in Wheat Plants

**Figure 1:**
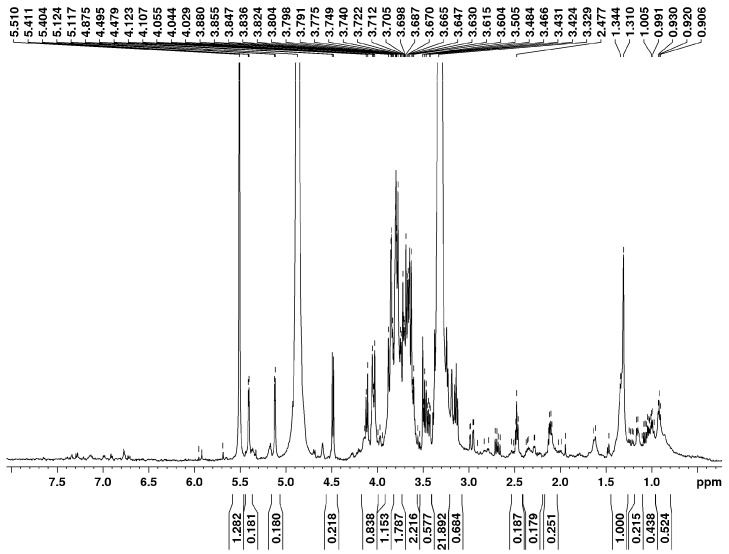
^1^H-nuclear magnetic resonance spectrum of 2-day-old wheat plants pretreated with 100 µM ibuprofen.

**Figure 2:**
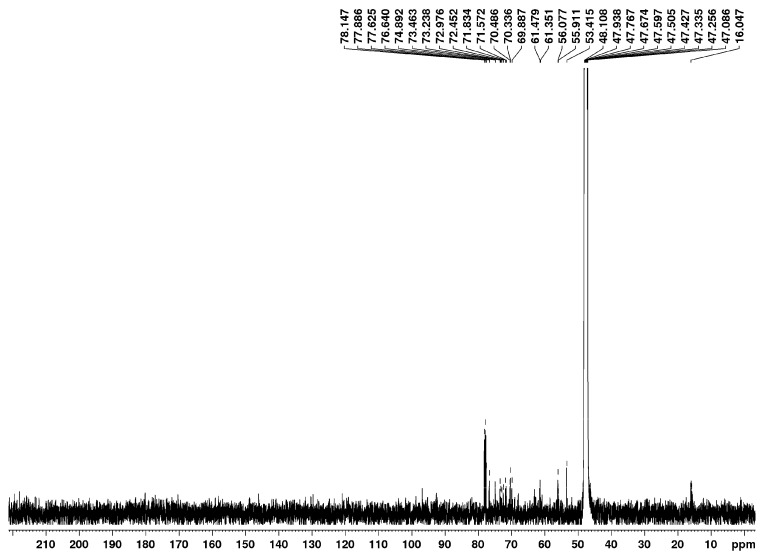
^13^C-nuclear magnetic resonance spectrum of 2-day-old wheat plants pretreated with 100 µM ibuprofen.

**Figure 3:**
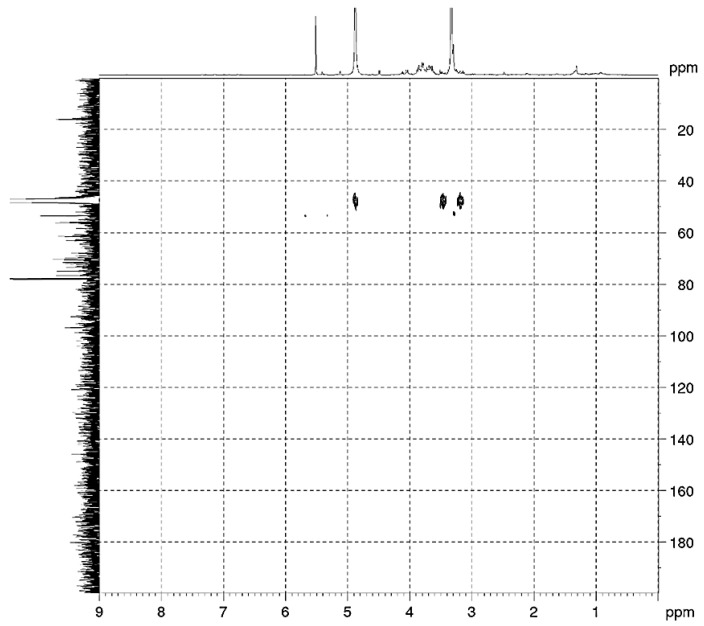
Heteronuclear multiple-bond correlation spectrum of 2-day-old wheat plants pretreated with 100 µM ibuprofen.

Structural analysis of the isolate by 1D and 2D NMR spectroscopy was conducted to confirm the absence of ibuprofen (IBU). The ^1^H and ^13^C NMR spectra (Figure 1 and Figure 2) show no characteristic signals in the aromatic (δ = 7.2–7.3 ppm) or carbonyl (δ ≥ 200 ppm) regions, respectively. Furthermore, the corresponding long-range correlations are absent in the HMBC (Heteronuclear Multiple-Bond Correlation) spectrum (Figure 3). These spectral data conclusively demonstrate that IBU is not present in the mixture.

### 2.2. Effect of IBU Pretreatment on Seed Germination and Growth of Wheat Seedlings Under Salt Exposure

Growth parameters are integral indicators of the physiological state of the plant. As well as a marker of the effectiveness of the use of various substances and the degree of exposure to various stress factors.

**Figure 4:**
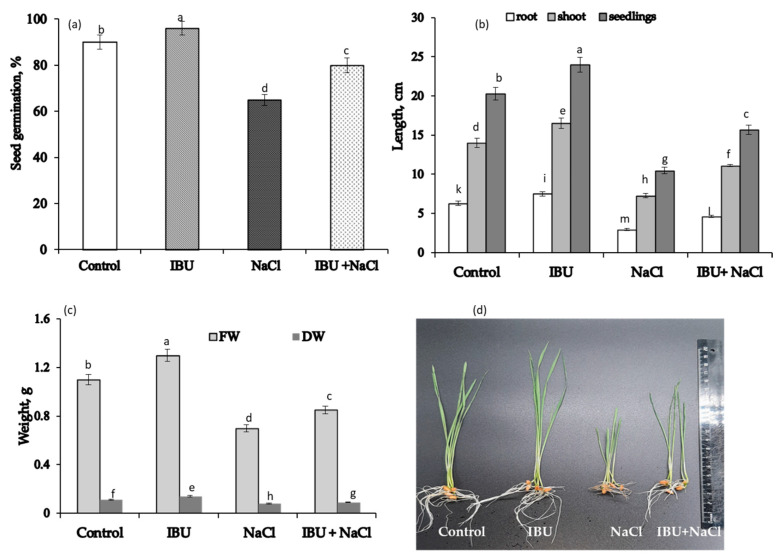
The effect of pre-sowing seed treatment with 100 μM ibuprofen on seed germination (**a**), seedlings length (**b**), fresh weight and dry weight (**c**) and visual appearance of 7-day-old wheat seedlings (**d**) under salinity conditions induced by 100 mM NaCl. Mean values and their standard errors are presented (*n* = 3). Different letters on the top of columns indicate statistically significant differences between the groups (*p* ≤ 0.05).

IBU seed pretreatment significantly enhanced germination energy by 7% (Figure 4a). This treatment effectively stimulated subsequent plant growth, increasing shoot and root length by 17% and 20%, respectively, resulting in a 20% greater total plant length compared to the control (Figure 4b). Consequently, pretreated plants accumulated 18% more fresh biomass and 7% more dry biomass (Figure 4c). Furthermore, they exhibited greater overall vigor and more advanced developmental morphology based on visual assessment (Figure 4d).

Germination under 100 mM NaCl stress significantly suppressed wheat seedling growth. Compared to the unstressed control, salt stress reduced germination energy by 28%, total plant length by 50%, and fresh and dry biomass by 36% and 27%, respectively (Figure 4a–c). The visual phenotype of the stressed plants clearly indicated a compromised physiological state (Figure 4d).

Pretreatment with IBU, however, markedly improved the physiological status of wheat plants subjected to salinity. When compared to the salt-stressed control plants without IBU pretreatment, the IBU-pretreated plants exhibited a significant mitigation of stress effects: the reduction in germination energy was attenuated to 11%, in plant length to 21%, and in fresh and dry biomass to 23% and 18%, respectively (Figure 4a–c). The improved visual phenotype of IBU-pretreated plants under stress further corroborated the finding that this treatment enhances wheat tolerance to saline conditions (Figure 4d).

### 2.3. The Effect of IBU Seed Pretreatment on Hormonal Balance of Wheat Seedlings Under Salinity

**Figure 5:**
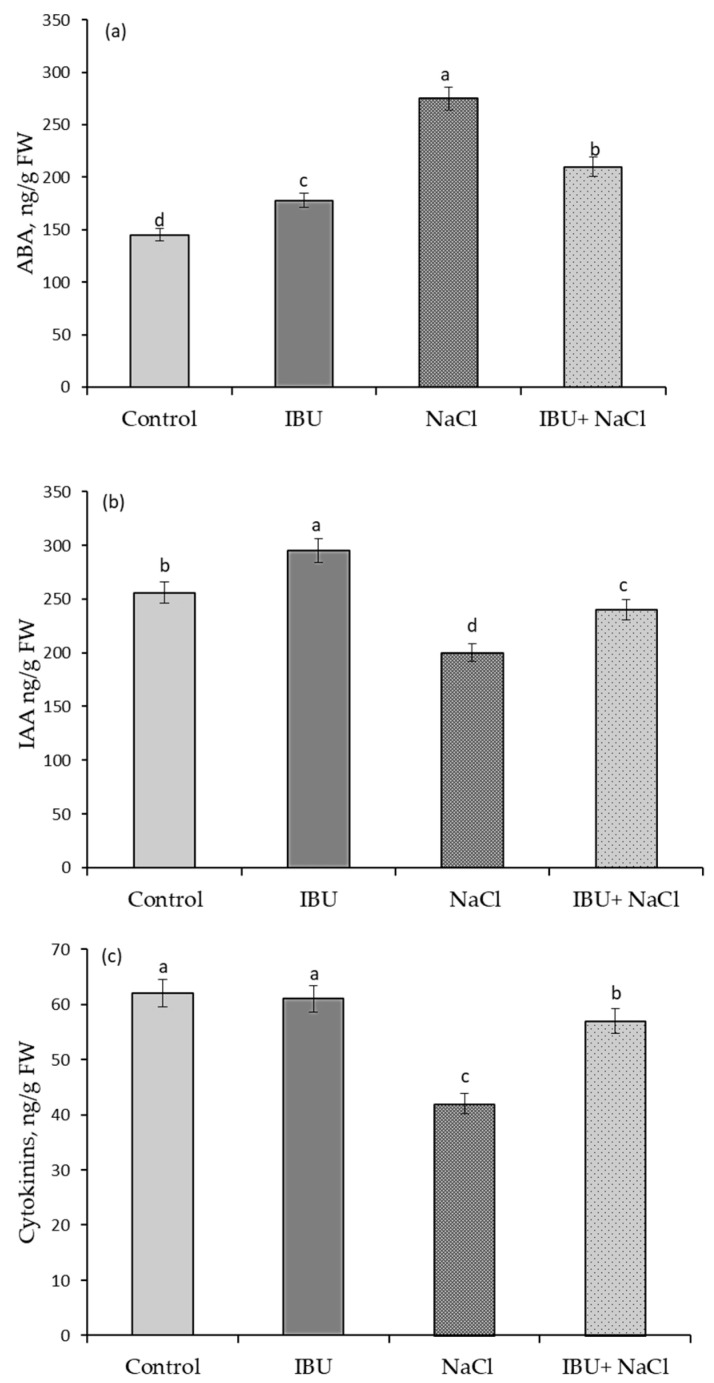
The effect of pre-sowing seed treatment with 100 µM ibuprofen on the contents of abscisic acid (**a**), indolylacetil acid (**b**), and cytokinins (**c**) in 7-day-old seedlings under salinity conditions. Mean values and their standard errors are presented (*n* = 3). Different letters on the top of columns indicate statistically significant differences between the groups (*p* ≤ 0.05).

The results of an enzyme-linked immunosorbent assay (ELISA) of abscisic acid (ABA), indolylacetil acid (IAA), and cytokinins (CKs) in IBU-pretreated 7-day-old wheat seedlings under normal and salinity conditions are presented in Figure 5.

Under physiological growth conditions, seed pretreatment with IBU led to an increase in ABA content by 23% and IAA content by 15%, while it did not affect the level of CKs. Exposure to stress factors typically causes significant shifts in phytohormone balance. Indeed, the presence of 100 mM NaCl resulted in a 90% accumulation of ABA and a decrease in IAA by 22% and CKs by 32% compared to the control.

Pretreatment with IBU under salinity conditions had a protective effect on the state of the hormonal system. This is indicated by data on the content of phytohormones in these plants. Specifically, the ABA content was 45% higher, and the IAA and CKs contents were 6% and 9% lower than the control level (Figure 5).

### 2.4. Seed Priming with Ibuprofen Modulates the Antioxidant Status in Wheat Seedlings

Under non-stress conditions, IBU pretreatment increased glutathione (GSH) by 11%, decreased oxidized glutathione (GSSG) by 13%, and ultimately raised the GSH/GSSG redox ratio by 28%. Furthermore, glutathione reductase (GR) activity showed a 10% increase over the control. Concomitantly, IBU application resulted in a balanced shift in ascorbate metabolism: a 12% rise in ascorbate (ASA) levels accompanied by a proportional 12% increase in ascorbate peroxidase (APX) activity. These plants also showed only a minor (8%) rise in H_2_O_2_, with malondialdehyde (MDA) levels remaining similar to the control (Table 1).

Salt stress (100 mM NaCl) triggered oxidative stress, leading to profound alterations in the redox homeostasis of plants. Exposure to NaCl resulted in an 89% surge in H_2_O_2_ production and a 67% increase in MDA content compared to control plants. Concurrently, the level of GSH dropped by 34%, whereas the GSSG rose by 60%. The GSH/GSSG ratio in these plants was 60% lower than that of the control. The level of ASA was reduced by approximately 50% in these plants. This redox shift was associated with a marked upregulation of key antioxidant enzymes: GR activity increased by 85%, and APX activity by 55%.

IBU-pretreatment exerts a protective effect on plant redox metabolism. This is supported by the following parameters. In treated plants, H_2_O_2_ content was 42% and MDA content was 33% above the control level. Compared with the control, the treated plants exhibited an 11% and 9% reduction in glutathione and ascorbate contents, respectively, and a 26% increase in GSSG levels. The GSH/GSSG ratio was 30% lower in these plants compared to the control. These changes were accompanied by the activation of GR by 32% and APX by 22% (Table 1).

### 2.5. Correlation Matrices

A factor analysis showed that all studied indicators (Table 2) could be divided into two clusters, with a mutual positive correlation within them and a negative correlation between them. The first cluster included the content of IAA and CKs, the levels of GSH and ASA, as well as the plant length parameter. The second cluster included the content of ABA and GSSG, the activity of GR and APX, as well as markers of oxidative stress (H_2_O_2_, MDA). These results indicate that the ability of IBU to modulate plant redox metabolism (Table 1) is closely associated with hormonal reprogramming (Figure 4), which in turn correlates with the effect of the treatment on plant growth parameters (Figure 3).

## 3. Discussion

The results of this study provide novel insights into the physiological and biochemical mechanisms underlying the beneficial effects of seed priming with ibuprofen (IBU) on wheat under saline conditions. The central finding is that the pronounced positive effects on growth and stress tolerance occur in the absence of accumulation of the xenobiotic in plant tissues. This key observation strongly supports the hypothesis that IBU acts as a hormetic preconditioning agent, triggering adaptive responses rather than exerting a direct toxic or residual effect.

In line with the first objective, our analytical data confirm the absence of IBU accumulation. This is a critical point, as it distinguishes the action of IBU from that of classical agrochemicals that often rely on persistence within the plant. This finding aligns with the broader concept of chemical priming, where the initial stimulus is transient, but the induced physiological state is lasting [17].

At the same time, the drug stimulates germination and positively affects the growth parameters of wheat (Figure 4). This is supported by literature data, which show that the presence of IBU in the seed germination medium stimulates the growth and development of the root system in lettuce, *Lactuca sativa* L. [18], and wheat, *Triticum aestivum* L. [19]. Most importantly, our results clearly demonstrate that IBU pretreatment not only stimulates growth under optimal conditions but also confers a distinct protective advantage, significantly enhancing wheat tolerance to salinity stress.

The coordinated regulation of cellular metabolism by phytohormones forms the basis of plant growth, development, and stress adaptation. Our data reveal that IBU specifically modulates this regulatory network by influencing the balance of abscisic acid (ABA) and indole-3-acetic acid (IAA), while cytokinin levels remained largely unaffected (Figure 5).

ABA is a versatile phytohormone with a dual role, integral to both fundamental developmental processes and as a master regulator of adaptive stress responses [7,20]. Consequently, the induction of ABA accumulation by IBU, a xenobiotic, is a significant and expected phenomenon that aligns with its observed protective function. This finding is corroborated by literature reporting that IBU triggers ABA accumulation in other plant species, such as malabar spinach (*Basella alba* L.) and duckweed (*Lemna minor* L.), where it is associated with enhanced stress tolerance or biomass production [21,22].

Simultaneously, auxin—predominantly in the form of IAA—orchestrates essential growth processes, including cell division, elongation, and organogenesis [23]. Our results demonstrate that under optimal conditions, IBU elevates IAA levels, which likely contributes to the observed growth stimulation. More critically, under salinity stress, IBU pretreatment played a protective role by attenuating the stress-induced decline in IAA content. This maintenance of auxin levels is crucial for sustaining growth under adverse conditions. Therefore, a central mechanism underlying the beneficial effects of IBU is its capacity to fine-tune hormonal balance. Under non-stress conditions, it elevates both ABA and IAA. Under salt stress, it mitigates hormonal dysregulation by preventing excessive ABA accumulation and maintaining IAA levels, thereby stabilizing the internal hormonal milieu and enabling sustained growth and adaptation (Figure 5).

In addition to hormonal regulation, the antioxidant system is fundamental for plant growth, development, and stress adaptation. Data on the capacity of IBU to affect plant redox metabolism remain scarce. For instance, IBU treatment was reported to stimulate the accumulation of glutathione (GSH) and the activity of glutathione S-transferase (GST) and peroxidase in malabar spinach (*Basella alba* L.) [21], while showing no effect on ascorbate (ASA), GSH, or the enzymes glutathione reductase (GR) and ascorbate peroxidase (APX) in crested wheatgrass (*Agropyron cristatum* L.) [24].

In contrast to these variable reports, our study revealed a coordinated upregulation of key components of the ascorbate–glutathione cycle [25] in wheat—specifically, increased levels of GSH, and ASA, along with enhanced activities of GR and APX (Table 1). This synchronized response strongly suggests that IBU seed priming boosts the overall antioxidant defense potential, preparing the plant for subsequent stress.

The priming of this antioxidant capacity may be initiated by a modest increase in H_2_O_2_ levels observed in IBU-pretreated plants under non-stress conditions. H_2_O_2_ can act as a signaling molecule, triggering downstream adaptive responses. Beyond their protective role, the antioxidants GSH and ASA, along with the regulated H_2_O_2_ pool, are essential for fundamental processes like mitosis, directly linking redox homeostasis to growth promotion [26].

Thus, these antioxidants contribute substantially to IBU’s effects in two ways: they support IBU-induced growth promotion and protect cellular membranes from salinity-induced oxidative damage. The upregulation of GR and APX activities indicates that IBU fine-tunes the cellular redox state. This precise control is crucial for maintaining the optimal balance of reactive oxygen species (ROS), GSH, and ASA—a balance essential for normal metabolic function under stress. This conclusion is directly supported by the greater stability of redox metabolism observed in IBU-pretreated plants under salinity stress compared to untreated controls. Key indicators of this enhanced stability include a more favorable GSH/GSSG ratio (indicative of a reduced cellular environment), lower levels of malondialdehyde (MDA, a marker of lipid peroxidation), and modulated H_2_O_2_ content (Table 1), which collectively correlate with improved growth parameters (Figure 4).

## 4. Materials and Methods

### 4.1. Seed Material, Pre-Sowing IBU-Treatment

The experiments were carried out on soft spring wheat seedlings (*Triticum aestivum* L., BBAADD 2n = 42, Ekada 70). Wheat seeds were obtained from Chishminsky Breeding Station, Ufa Federal Research Centre, Russian Academy of Sciences (Ufa, RB, Russia). The seeds were sterilized by immersing them in 96% ethanol for 60 s. Subsequently, they were washed with sterile water until the smell of alcohol disappeared. To soak the seeds, a solution of ibuprofen (Sigma-Aldrich, Burlington, MA, USA) [IBU: (C_13_H_18_O_2_)] was utilized. The seeds were kept in a solution of 100 μM IBU or water (Control) for 3 h. After soaking the seeds were dried in a desiccator with CaCl_2_ prior to planting.

### 4.2. Plant Material and Growth Conditions

Seeds pretreated and untreated with 100 μM IBU were grown in Petri dishes (on filter paper moistened with water- (Control) and 100 mM NaCl (Salinity) [8] by a 16/8 h light/dark photoperiod regime under (200 μmoL/(m^2^ s) at 22–24 °C for 3 days. After this, 3-day-old seedlings were transplanted into glasses with a 10% Hoagland’s solution with 100 mM NaCl solution and grown up to the age of 7 days. Control plants were grown in a nutrient solution without the addition of sodium chloride. Samples were collected at day 7 of germination, fixed in liquid nitrogen, and stored at −70 °C for further evaluation of physiological and biochemical parameters.

To analyze IBU content, seedlings IBU-treated were collected on the second day of germination. The seedlings were separated from the endosperm, immediately flash-frozen in liquid nitrogen, and stored at −70 °C until analysis.

### 4.3. Determination of Plant Growth Parameters

The intensity of growth processes (seed germination, length, fresh and dry weight) was assessed according to [27]. Each variant was carried out in three replicates, with 30 plants per replicate.

### 4.4. Ibuprofen Extraction and Quantification

Ibuprofen was extracted from plant tissues according to the protocol described by [28,29]. The frozen plant materials (ca. 1 g) were chopped and triturated. The obtained probe was placed in a polypropylene tube and treated with 0.5 mL of HCl 0.1 M and 4 mL of methanol (MeOH). Then, the tube was sonicated for 10 min and centrifuged for 15 min at 4000 rpm. This process was repeated three times. So, the extracts were mixed to obtain an approximate volume of 12 mL that was evaporated to 4 mL. Then, the obtained product was diluted to 200 mL with Milli-Q water in a volumetric flask and passed under vacuum through Oasis HLB cartridges (3 cc/60 mg, Waters, Medford, MA USA). Once the sample passed, the cartridges were air-dried. Further the analytes were eluted with 10 mL of methanol using a vacuum first and then under gravity. The final extract was made up to exactly 1 mL with 2% MeOH by weight. Nuclear magnetic resonance spectroscopy was performed on a Brucker Avance 500. The ^1^H NMR spectrum was recorded at 500 MHz and ^13^C-{^1^H} NMR spectra at 125 MHz in MeOD. Homo- and heteronuclear shift correlation experiments (COSY, HSQC, HMBC, NOESY) were carried out according to standard Bruker procedures. The chemical shifts are reported in ppm relative to tetramethylsilane (TMS) as the internal standard.

### 4.5. Determination of Non-Enzymatic Antioxidants

#### 4.5.1. Determination of Reduced Glutathione (GSH) and Oxidized Glutathione (GSSG) Content

Levels of GSH and GSSG were determined using o-phthalaldehyde (79760, Sigma-Aldrich, Burlington, MA, USA). Plant samples (approximately 0.5 g) were homogenized in 4 mL of a mixture consisting of 0.1 M potassium phosphate buffer (pH 8.0) and 25% metaphosphoric acid (HPO_3_) solution in a ratio of 3.75:1 (by volume) as recommended in [30]. The homogenate was centrifuged for 10 min at 8000× *g*, and then, the supernatant was centrifuged again for 5 min at 13,000× *g*. A 0.5 mL aliquot of the supernatant was used to analyze the content of reduced and oxidized glutathione according to [30]. To assess the GSH and GSSG contents, the kinetics of the fluorescence intensity of the formed complexes were recorded at pH 8.0 and pH 12.0, respectively, using an EnSpire Model 2300 Multilabel Microplate Reader (PerkinElmer, Boston, MA, USA) at 420 nm (excitation wavelength 350 nm). The levels of glutathione forms were expressed in μmoL/mg protein. The GSH/GSSG ratio, indicating plant redox status, was measured as total glutathione (GSH + GSSG)/oxidized glutathione (GSSG). 

#### 4.5.2. Ascorbate (ASA) Content

The amount of ASA was determined by the titration method [31]. The wheat samples (10 g) were crushed in a porcelain mortar, extracted with 10 mL of distilled water, agitated, and filtered through a paper filter. An amount of 20 mL of filtrate was taken into a conical flask and 1 mL of 2% hydrochloric acid (HCl), 0.5 mL of 1% potassium iodide (KI), and 2 mL of 0.5% starch were added, and then stirred. The resultant mixture was titrated with 0.001 molL^−1^ potassium iodate (KIO_3_) until it reached a stable blue color staining. The concentration of ASA was reported as mg% FW [30].

### 4.6. Measurement of the Enzymatic Antioxidants

#### 4.6.1. Glutathione Reductase (GR) Activity

Glutathione reductase (GR, EC 1.6.4.2) catalyzes the reduction of GSSG to GSH using NADPH as the electron donor. Enzyme activity is determined spectrophotometrically by monitoring the oxidation of NADPH to NADP^+^, indicated by a decrease in absorbance at 340 nm [32]. Seedlings (0.25 g) were ground in liquid nitrogen by adding 0.75 mL of 50 mM Tris-HCl buffer (pH 7.6). The homogenate was centrifuged at 22,000 *g* at 4 °C for 30 min and the supernatant was used for enzyme assay. The reaction mixture contained 50 mM Tris-HCl buffer (pH 7.6) (1.91 mL), 0.15 mM NADPH (0.02 mL) and enzyme extract (0.05 mL). Before measurement, 1 mM GSSG (0.02 mL) was added to the experimental cuvette (final assay volume 2 mL). The reaction was monitored by a decrease in absorbance of NADPH at 340 nm [32].

#### 4.6.2. Ascorbate Peroxidase (APX) Activity

The activity of ascorbate peroxydase (APX, EC 1.11.1.11) was estimated by monitoring the oxidation of the ascorbate at 290 nm [32]. The reaction mixture (2.93 mL) consisted of 50 mM phosphate buffer (pH 7.0), (0.03 mL) 17 mM ASA, (0.03 mL) EDTA, 0.01 mL extract. The response started with the addition of 0.03 mL of 0.06% H_2_O_2_ and was determined in the first 100 s. The data received were expressed as μmoL ascorbate oxidized/mg protein min.

The activity of antioxidant enzymes was determined spectrophotometrically and normalized to the total protein content in the sample. Protein concentration was measured using the Bradford method [33] with bovine serum albumin (BSA) as a standard. All spectrophotometric measurements were performed on a UNICO 2800 instrument (United Products & Instruments, Middlesex, NJ, USA).

### 4.7. Immunoassay of Phytohormones

The contents of abscisic acid (ABA), indoleacetic acid (IAA) and cytokinins (CKs) were measured in 10 wheat seedlings (1–1.5 g of fresh weight) by enzyme-linked immunosorbent assay (ELISA) using specific polyclonal rabbit antibodies against each of the investigated compound and anti-rabbit antibodies labeled with peroxidase as described earlier [7]. Plant material was homogenized in 80% ethanol (1:10, *w*/*v*) and kept at 4 °C for 16 h to extract phytohormones. CKs were immunoassayed in aqueous residues of ethanol extracts. We have determined the total content of zeatin derivatives (zeatin, its riboside, and nucleotide) immunoreactive in the test system with rabbit antibodies to zeatin riboside [7]. For ABA and IAA measurement, the remaining aqueous residue was acidified with HCl to pH 2.5 and partitioned twice with diethyl ether. After that, ABA and IAA were transferred from the organic phase into 1% sodium hydrocarbonate (pH 7–8), re-extracted with diethyl ether, methylated with diazomethane, and immunoassayed using polyclonal antibodies to ABA and IAA. The quantity of phytohormone-specific antibodies immobilized on the plate was determined using peroxidase-conjugated anti-rabbit antibodies (N. F. Gamaleya National Research Center, Moscow, Russia). Peroxidase activity was measured as the absorbance at 492 nm of the ortho-phenylenediamine chromogenic product, using the Benchmark Microplate Reader (Bio-Rad, Hercules, CA, USA) [7].

### 4.8. Oxidative Stress Markers

To determine malondialdehyde (MDA) [31], wheat seedlings were ground in distilled water and then homogenized in 20% trichloroacetic acid. The homogeneous samples then were centrifuged (10,000× *g*, 10 min). Then, the supernatant was mixed with 0.5% thiobarbituric acid prepared in 20% trichloroacetic acid and was kept in a boiling water bath (100 °C, 30 min), and quickly cooled. Absorbance was spectrophotometrically (SmartSpecTM Plus, Bio-Rad, Hercules, CA, USA) measured at 532 nm and 600 nm. The MDA was expressed as nmoL/g FW.

To determine H_2_O_2_ [34], samples of plant material homogenized (1:5 weight/volume) in 0.05 M sodium phosphate buffer (FB), pH 6.2. The supernatant was separated by centrifugation (Eppendorf^®^ Microcentrifuge 5415 R, Humburg, Germany) at 15,000× *g* for 15 min. The concentration of H_2_O_2_ in the supernatant was spectrophotometrically (SmartSpecTM Plus, Bio-Rad, Hercules, CA, USA) determined using xylenol orange in the presence of Fe^2+^ at 560 nm. The H_2_O_2_ was expressed as µmoL/g FW.

### 4.9. Statistical Analysis

All physiological, biochemical and physicochemical experiments were performed on three biological and three analytical replicates. The experimental data were subjected to a one-way analysis of variance (ANOVA) using SPSS 13.0 software for Windows (SPSS Inc., Chicago, IL, USA). The significant differences between mean values were estimated using the least significant difference (LSD) test at *p* ≤ 0.05. The data in the figures and table are presented as mean values and their standard errors (±SE). Correlation analyses (Pearson’s coefficients and matrix construction) were performed using STATISTICA 10.0.

## 5. Conclusions

This study demonstrates that priming wheat seeds with 100 µM ibuprofen (IBU) is an effective strategy for enhancing salinity tolerance. Despite the absence of xenobiotic accumulation in seedling tissues, the treatment exerts a pronounced dual beneficial effect, promoting growth under optimal conditions and providing protection under stress. The primary mechanism underlying this action is IBU’s capacity to finely modulate the plant’s physiological state by regulating phytohormone balance (abscisic acid, indoleacetic acid and cytokinins) and activating the antioxidant system (the ascorbate–glutathione cycle). Under salt stress, IBU priming mitigates characteristic stress-induced shifts, maintaining a more balanced hormonal and redox status, which ultimately translates into improved seedling vigor and growth performance. These findings position IBU in a novel role—as a chemical priming agent of a hormetic nature, whose efficacy is based on the activation of the plant’s endogenous regulatory and defense systems. This offers promising avenues for developing new, sustainable agrochemical approaches.

## Figures and Tables

**Table 1:** Influence of 100 µM ibuprofen on the content of GSH, GSSG, the GSH/GSSG ratio, ASA, MDA, H_2_O_2_ and the state of antioxidant enzymes, GR and APX, in 7-day-old seedlings under salinity conditions (100 mM NaCl).

Treatment	GSH, µmoL/mg Protein	GSSG, µmoL/mg Protein	GSH/ GSSG	ASA, mg % FW	GR, nmoL/mg Protein min	APX, μmol Ascorbate Oxidized/ mg Protein min	H_2_O_2,_ µmoL/g FW	MDA, nM/g FW
Control	18 ± 0.72 ^b^	1.5 ± 0.06 ^c^	12 ± 0.48 ^b^	4.2 ± 0.17 ^b^	4.0 ± 0.16 ^d^	0.9 ± 0.04 ^d^	4.8 ± 0.19 ^d^	49 ± 1.96 ^d^
IBU	20 ± 0.8 ^a^	1.3 ± 0.05 ^d^	15.4 ± 0.6 ^a^	4.7± 0.18 ^a^	4.4 ± 0.18 ^c^	1.0 ± 0.04 ^c^	5.2 ± 0.2 ^c^	50 ± 2.0 ^c^
NaCl	12 ± 0.48 ^d^	2.4 ± 0.09 ^a^	5.0± 0.2 ^d^	2.2 ± 0.08 ^d^	7.4 ± 0.29 ^a^	1.4 ± 0.06 ^a^	9.1 ± 0.36 ^a^	82 ± 3.28 ^a^
IBU + NaCl	16 ± 0.64 ^c^	1.9 ± 0.08 ^b^	8.4 ± 0.34 ^c^	3.8 ± 0.12 ^c^	5.3 ± 0.25 ^b^	1.1 ± 0.05 ^b^	6.8 ± 0.31 ^b^	65 ± 2.84 ^b^

The data shown are the mean values and their standard errors (*n* = 3); the averages with different letters are significantly different (*p* ≤ 0.05).

**Table 2:** Correlation matrix representing the correlation between key parameters examined in this study.

	ABA	IAA	CKs	GSH	GSSG	GSH/GSSG	ASA	GR	APX	H_2_O_2_	MDA	Length
**ABA**	1											
**IAA**	−0.78770466	1										
**CKs**	−0.9589621	0.86668239	1									
**GSH**	−0.8837946	0.98381872	0.938403	1								
**GSSG**	0.91261145	−0.96901669	−0.93813	−0.9947363	1							
**GSH/GSSG**	−0.8316871	0.98616267	−0.864091	0.98177389	−0.983851862	1						
**ASA**	−0.89649495	0.95800355	0.970897	0.98828285	−0.976007292	0.941374	1					
**GR**	0.98678048	−0.85748113	−0.9909	−0.9357687	0.948807919	−0.87714	−0.95435	1				
**APX**	0.99376992	−0.81210511	−0.98361	−0.9035079	0.921447843	−0.83798	−0.92758	0.996556	1			
**H_2_O_2_**	0.98687687	−0.87684007	−0.97522	−0.9475444	0.966270819	−0.90775	−0.95048	0.994703	0.9882581	1		
**MDA**	0.97477976	−0.9033993	−0.9664	−0.9631408	0.980715131	−0.93416	−0.95748	0.986945	0.9754262	0.997574	1	
**Length**	−0.8569764	0.99038432	0.901216	0.99461155	−0.992778292	0.996157	0.967296	−0.9065	−0.869959	−0.92847	−0.950224	1

## Data Availability

The original contributions presented in the study are included in the article, further inquiries can be directed to the corresponding author.
